# The Impact of Interpersonal Interaction on Purchase Intention in Livestreaming E-Commerce: A Moderated Mediation Model

**DOI:** 10.3390/bs14040320

**Published:** 2024-04-12

**Authors:** Shuai Ling, Can Zheng, Dongmin Cho, Yonggu Kim, Qizhen Dong

**Affiliations:** 1 Department of Design and Manufacturing Engineering, Jeonbuk National University, Jeonju 54896, Republic of Korea; shuailing@jbnu.ac.kr; 2 Department of Industrial Design, Jeonbuk National University, Jeonju 54896, Republic of Korea; mellgipson@jbnu.ac.kr (D.C.); nine221@jbnu.ac.kr (Y.K.); 3 Department of Hospitality and Tourism Management, Sejong University, Seoul 05006, Republic of Korea; dongqizhen@sju.ac.kr

**Keywords:** livestreaming e-commerce, purchase intention, interpersonal interaction, brand identification, psychological distance, time pressure

## Abstract

Over the last few years, livestreaming e-commerce has shown rapid growth and has become an important form of e-commerce. However, the potential mechanisms of interpersonal interaction’s influence on purchase intention in livestreaming e-commerce have yet to be fully investigated. Based on the SOR (Stimulus-Organism-Response) framework, this study reveals the association between interpersonal interaction (consumer–anchor interaction and consumer–consumer interaction), psychological distance, consumer purchase intention, and the positive role of brand identification and time pressure in this context of influential relationships. The results of analyzing 603 questionnaires show that psychological distance between consumers and products plays a mediating role in the effect of interpersonal interaction on purchase intention. Meanwhile, this study found that consumers’ brand identification with the products in the live room was effective in enhancing the direct effect of interpersonal interaction in the model. Additionally, the time pressure associated with limited-time sales was also found to be effective in enhancing the effects of interpersonal interaction and psychological distance on purchase intention. The results of this study reveal the potential influence mechanisms of interpersonal interactions with various identities in livestreaming e-commerce, providing theoretical guidance and practical insights for practitioners in the field.

## 1. Introduction

As communication and logistical technology advances and the continuous integration of industries and supply chains continues, livestreaming e-commerce (LSE) has emerged as a novel business strategy that promotes a rise in consumption [1]. LSE can effectively reduce consumer search costs and provide consumers with immersive consumption scenarios to enhance their consumption experience [2]. The U.S. online commerce market size has grown from USD 6 billion in 2020 to USD 17 billion in 2022 and is expected to reach USD 35 billion in 2024, while in Europe, 40% of consumers indicate that livestreaming e-commerce platforms can evoke their shopping interest [3]. According to the latest data from the Chinese Academy of Metrology and Science, in 2022, the cumulative value of livestreaming on the key monitored e-commerce platforms exceeded CNY 120 million, with a cumulative viewing value of over 1.1 trillion and more than 95 million livestreaming products. During the first half of 2023, focused monitoring of e-commerce platforms revealed a cumulative livestreaming sales volume of CNY 1.27 trillion, with over 110 million livestreaming sessions and over 70 million livestreamed products [4]. This demonstrates a rapidly growing trend in LSE.

The development of LSE has rapidly gained widespread attention by the academic community, and existing studies have concluded that LSE outperforms traditional e-commerce in many ways. For example, the visual effects, sociality, entertainment, and immersive experience are characteristics of LSE that can positively influence consumer purchase intention [5,6]. The anchor, on the other hand, can fully present the information and functions of the product through the introduction and demonstration of their use, thus promoting consumer participation [7]. This objective shopping environment is also conducive to enabling the anchor to generate trust in the products in the live room by building trust with consumers [8]. The interactivity brought by LSE in the form of livestreaming is a significant competitive advantage in comparison to more traditional e-commerce [9,10]. Meanwhile, related studies have concluded that interpersonal interaction is an important way of establishing good relationships with consumers, enhancing product image, and effectively influencing consumer purchase intention [11,12,13]. Therefore, interpersonal interaction should be the focus of attention in all LSE platforms. In this regard, gaining insights into how interpersonal interactions impact consumer purchase intention is essential for LSE to enhance its market competitiveness.

The impact of interpersonal interaction in the context of LSE has attracted considerable scholarly interest. Shiu et al. emphasized that interaction in livestreaming can favorably impact the immersive experience and promote purchase intention [14]. Kang et al., argued that interactivity enhances consumers’ interpersonal intimacy and engagement in LSE [9]. Zhou et al., argued that the anchor’s interaction can enhance the effect of live broadcasting and positively impact the live room sales [15]. Liu et al., emphasized that interactivity is one of the main features of LSE and argued that interaction can significantly affect the consumer’s mind-streaming experience and trust, thus affecting purchase intention [16]. These studies inspired the thought behind this study. However, they did not comprehensively consider the actual situation of the live room, and most of them made interpersonal interaction a single dimension to be studied, ignoring the interaction between people with different identities.

Previous studies have also ignored the impact of changes in the cognitive capacities of consumers and the different forms of live broadcasting. In LSE, anchors make consumers quickly familiar with the products and make their descriptions and perceptions of the live room products more concrete by continuously showing them the products, describing the specific information of the products, and answering consumers’ questions about the products. Existing studies have shown that the degree of abstraction or concreteness of an individual’s description of a thing also affects how far or near they are in terms of psychological distance from that thing [17,18]. Therefore, the component of psychological distance was included in this study. Brand-exclusive and limited-time sales, as the main forms of LSE [1,19], should be fully considered when conducting related studies. Branded specials are usually brand-led and focus on showcasing products from a specific brand. The aim of limited-time sales is to emphasize the urgency of time and the limited quantity of products. Therefore, brand identification and time pressure in this context are included in the framework of this study. Brand identification is the degree to which some consumers who prefer a particular brand identify with that brand [20]. This determines how specific the brand is for consumers [21]. Time pressure is a subjective sense of urgency and anxiety consumers perceive during the purchase process [22]. The difference between brand identification and time pressure lies in the fact that brand identification is the sense of belonging to a brand and is generated by consumers based on their previous experiences and perceptions [23], and this psychological state exists as the consumer’s “inner”, while time pressure is the “outer” influence of the external environment on consumers. No research so far integrates the effects of interpersonal interaction, brand identification, psychological distance, and time pressure on the purchase intention of LSE. Therefore, by integrating the above theories, this study aims to establish a model under the integrated perspective of “inner” and “outer” to fill the gaps in relevant research. The objective of this study is to elucidate the following research questions:

(1) How does interpersonal interaction affect consumer psychological distance and purchase intention for LSE products?
(2) What role does psychological distance play in the influence of interpersonal interaction on consumer purchase intention?
(3) What roles do brand identification and time pressure play in the interpersonal interaction–psychological distance–purchase intention research framework?

This study presents a novel theoretical framework for consumer psychology in the LSE, which can help relevant practitioners better understand how interpersonal interaction affects purchase intention by influencing psychological distance. This research also uncovers the moderating effect of time pressure and brand identification in this influential relationship and provides useful insights on how to better promote the impact of LSE on purchase intention. This fills the gap in the related research and provides theoretical guidance and practical insights for LSE platforms.

The structure of this paper is as follows. First, a literature review examines the relevant theories and the proposal of hypotheses and models. Subsequently, the research methodology is presented, the empirical process is described, the results are discussed, and conclusions are drawn. Finally, limitations and future research are put forth.

## 2. Literature Review and Hypothesis Development

### 2.1. Literature Review

#### 2.1.1. Interpersonal Interaction

The concept of “Interaction” was first proposed by Georg Simmel, a German sociologist, and describes the mutual influences and effects between individuals or groups through exchanges [24]. Georg Simmel considered cooperation and conflict, leader and follower, and interpersonal exchange processes as forms of interpersonal interaction [25]. With the gradual popularization of computers, the concept of interaction has been applied to human–computer interaction. For example, Steuer argues that interaction can be defined from the standpoint of user control over the medium, referring to the degree to which users can actively modify the content and format of messages in the medium in real-time [26]. Based on this, scholars have segmented the types of interaction. For example, Hoffman et al. categorize interactions into human–computer interactions (focusing on interactions referring to human–media interactions) and interpersonal interactions (focusing on human–human interactions) [27]. Nambisan et al. added the dimension of product interaction on top of this [28]. McMahan proposed categorizing interactions into three types: interactions between individuals, between individuals and computers, and between individuals and content [29]. In the research on interpersonal interaction (human-to-human interaction), scholars have mainly divided interaction, depending on its focus, into two categories: information perception and degree of interaction. The dimensions of interpersonal interaction in information perception are usually divided into perceived expertise, similarity, familiarity, and likability [30]. On the other hand, the degree of interaction is divided according to the communication between different populations during the interpersonal interaction, such as consumer–anchor and consumer–consumer [13,31]. Because the anchor display area and comment area are the main sections of the livestreaming page, this study mainly focuses on the interactions between different populations, and interpersonal interactions are divided into consumer–anchor interaction (CAI) and consumer–consumer interaction (CCI). CAI and CCI are defined as anchor–consumer and consumer–consumer communication about products and emotions in LSE.

#### 2.1.2. Psychological Distance

The concept of psychological distance was first introduced by the British aesthetician Bullough in art appreciation and then by Liberman et al. in social psychology [32,33]. The construal level theory typically uses the term psychological distance to unify the factors that influence the level of construal [34]. More specifically, when psychological distance is high, individuals tend to adopt high-level explanations that characterize the essence and overall features of abstraction. When the opposite is true, individuals tend to adopt low levels of explanation, characterizing concrete surfaces and local features [35]. Psychological distance has been given different meanings as research has delved deeper into the study. Trope et al. defined it as the perceived proximity of something to the self at a given moment [36]. Kim et al. defined psychological distance as the distance the audience perceives between events in their mental space [37]. This study defines psychological distance as the self-centered perception of the proximity of products in LSE, which is based on psychological estrangement or proximity.

#### 2.1.3. Brand Identification

Brand identification depends on the consumer’s association with the brand and is one of the five core components of the brand relationship [38]. Research in social identity theory suggests that consumers usually exhibit a social identity that goes beyond their own identity and is used to reflect their sense of self [39]. Consumer perception of brand uniformity is an effective way of embodying social identity [40]. In that regard, scholars usually understand the concept of brand identification from the perspective of social identity [41]. Therefore, brands are special social categories that consumers identify with [42], and consumers can utilize consumer brands to position their social identity [43]. Brand identification is frequently conceptualized as the extent to which a consumer’s self-image aligns with that of the brand or the consumer’s perceived belongingness to the brand [41,44]. Brand identification occurs when a brand is associated with consumer characteristics [40]. Summarizing the above, this study integrates the current LSE context and delineates brand identification as the extent of consumer alignment with the brand of products marketed within live rooms. This alignment encompasses the consumer’s sense of affiliation with the brand and the similarity of characteristics.

#### 2.1.4. Time Pressure

The decision-making process requires sufficient time, and time pressure may arise when the decision-making period is shorter than what the decision-maker requires [45]. This time pressure refers to the decision maker’s sense of urgency due to time not being sufficient, or anxiety due to the task not having been completed on time [46,47]. The subjective factor of time pressure in consumer scenarios comes from the discount rate of products, and the objective factor comes from the time constraints involved in the process, which constitute the opportunity cost and ultimately affect the consumer’s decision [48]. Therefore, time pressure is an important situational variable influencing consumer decision-making [49,50]. For example, Spears investigated the impact on purchase intention by analyzing the effect of time pressure on information processing [51]. Peng et al. investigated consumer behavior in social e-commerce by analyzing the moderating effect of time pressure in that context [22]. Academics have diverse perspectives on the impact of time pressure, with most considering both behavioral and emotional aspects. Summarizing the above discussion, this study incorporates the current state of the LSE and defines time pressure as the sense of urgency experienced by consumers due to limited-time offers or restricted sales.

### 2.2. Hypothesis Development

#### 2.2.1. Interpersonal Interaction and Purchase Intention

Prior research has demonstrated that consumer purchase intention is notably impacted by interaction [52,53]. Interpersonal interaction is a subset of the interaction concept and has experienced broader expansion and extension due to the evolution of online socialization [54]. Interaction on the web is characterized by high frequency, high information content, and low cost [55]. In LSE, this is due to the real-time, two-way mode of information exchange between buyers and sellers. In LSE, the display and the actions of anchors such as displays, question and answer periods, and personalized recommendations, along with the consumer’s communication process, contribute to consumers perceiving authenticity, visibility, and real-time characteristics [56]. This interaction allows for a closer connection between consumer–anchor and consumer–consumer interactions and increases their intimacy and trust [57,58,59]. Meanwhile, parasocial interaction theory suggests that their imagined intimacy influences consumer purchasing behavior [60]. Existing research also suggests that interaction can positively influence consumer behavior [61]. Therefore, this study anticipates that interpersonal interaction in LSE is positively related to consumer purchase intention. The hypothesis is as follows:

**Hypothesis 1a (H1a).**  *The higher the degree of CAI in LSE, the higher the consumers’ purchase intentions*.

**Hypothesis 1b (H1b).**  *The higher the degree of CCI in LSE, the higher the consumers’ purchase intentions*.

#### 2.2.2. Interpersonal Interaction and Psychological Distance

Online interactions are characterized by personalization and responsiveness [58]. Personalization in LSE stems from the anchor’s response to questions and requests from consumers, and the nature of this response is a tailored interaction for the consumer. On the other hand, existing research suggests that such personalized messages enable consumers to reduce uncertainty about the products and decrease psychological distance [62]. Responsiveness is an important factor in online interaction [63]. Responsiveness in LSE comes from the efficient collaboration and participation introduced by the live broadcasting characteristics. This high level of connection and interaction, when consumers use LSE, accelerates the consumption of information about the products by the consumers so that the impression of the products is more concretized, thus ultimately reducing the psychological distance between the consumers and the products. Meanwhile, studies have indicated that in the context of LSE, the anchor’s professional explanations and prompt responses foster consumer trust in the anchor’s credibility, enhance the sense of intimacy, and diminish the psychological distance between the products and consumers [58]. In addition, consumers can glean more details about the products and alleviate their perception of the products’ risks by engaging in discussions with other consumers in the live room, thereby reducing the psychological distance between the products and the consumers. Therefore, this study anticipates that interpersonal interaction within LSE will result in a diminished psychological distance between consumers and products. The hypothesis is as follows:

**Hypothesis 2a (H2a).**  *The higher the degree of CAI in LSE, the lower the psychological distance between consumers and products*.

**Hypothesis 2b (H2b).**  *The higher the degree of CCI in LSE, the lower the psychological distance between consumers and products*.

#### 2.2.3. Psychological Distance and Purchase Intention

Psychological distance has been extensively employed in consumer behavior research and recognized as a significant determinant of consumer purchase intention across different situations [64]. Existing studies indicate that psychological distance can alter an individual’s cognition of a particular object and ultimately influence consumer behavior [65,66]. Meanwhile, consumers usually focus on information similar to their construal level when making decisions [67]. Specifically, a closer psychological distance to the products reduces consumers’ sense of crisis and defensive behaviors. Additionally, it enhances their experience and ultimately increases consumers’ purchase intentions [58,62]. At the same time, a closer psychological distance to a product enhances consumers’ pro-social behaviors [68]. It encourages empathy among viewers during live broadcasts [69], e.g., consumers may show concern and support for a product, which increases their tendency to purchase the product. Whether it is a decreased crisis awareness or increased empathy, the closer psychological distance to the commodity leads to a more positive emotional connection for the consumer. Therefore, this study anticipates that the closer the psychological distance of consumers to the products in LSE is, the higher their purchase intention will be. The hypothesis is as follows:

**Hypothesis 3 (H3).**  *The lower the psychological distance between consumers and products in LSE, the higher the consumers’ purchase intentions*.

#### 2.2.4. The Mediating Effect of Psychological Distance

By summarizing the above hypotheses, this study concludes that in CAI, the anchor’s detailed, professional explanations and timely replies can reduce consumers’ psychological distance from the products by increasing intimacy and trust [58], resulting in a positive emotional connection to the products and ultimately influencing their purchase intention [68]. In CCI, details about products obtained from chats with other consumers can reduce the psychological distance between them and the products, by reducing the perceived risk of the products [58]. This results in a reduced sense of crisis and defensiveness and it ultimately influences their purchase intention [62]. Therefore, this study supports the idea that psychological distance mediates between interpersonal interaction and consumers’ purchase intentions. The hypothesis is as follows:

**Hypothesis 4a (H4a).**  *Psychological distance has a mediating role between CAI and consumer purchase intention*.

**Hypothesis 4b (H4b).**  *Psychological distance has a mediating role between CCI and consumer purchase intention*.

#### 2.2.5. The Moderating Effect of Brand Identification

Existing research suggests that two main mechanisms motivate consumers to develop brand identification. According to the first mechanism, consumers are motivated by the need for brand consistency [70], and according to the latter, consumers are motivated by their need to nurture their self-esteem [71]. Each of these two mechanisms belongs to a different part of social identity. Consistency-related research suggests that consumers look for brands that share the same attributes as their own [72]. This brand consistency leads consumers to feel a sense of belonging and ultimately develops brand identification [44]. Self-esteem-related research suggests that consumers enhance their self-image by purchasing well-known brands [70], and the same result is also reflected in research related to organizational identity [73]. This indicates that consumers who exhibit brand identification possess a self-image that aligns with the brand and also perceive that the brand enhances their image. This is related to the psychological distance of the consumers because a lower psychological distance implies that something is highly related or similar to an individual’s self-identity [74]. The enhancement of self-image implies that consumers are inclined toward their purchase intention [75,76]. In LSE, brand and other product information are continuously transmitted along with interpersonal interaction, which undoubtedly strengthens the psychological distance and tendency in purchase intention that brand identification brings to consumers. Therefore, the process of the impact of interpersonal interaction on psychological distance and consumer purchase intention in LSE can be influenced by the level of brand identification. Based on this, the present study posits that consumers hold varying perceptions of brand identification and differ in their level of brand consistency, and that they then have different levels of concern for psychological distance and purchase intention. When brand consistency is high (brand identification is high), interpersonal interaction is more likely to bring consumers closer to the products’ psychological distance and purchase intention. In contrast to that, when brand consistency is low (brand identification is low), the impact of interpersonal interaction is relatively small. Therefore, this study supports the idea that the effects of interpersonal interaction on psychological distance and consumer purchase intention are moderated by brand identification. When brand identification is high, the influence relationship is stronger; when brand identification is low, the influence relationship is weaker. The hypothesis is as follows:

**Hypothesis 5a (H5a).**  *Brand identification positively moderates the relationship between CAI and psychological distance*.

**Hypothesis 5b (H5b).**  *Brand identification positively moderates the relationship between CCI and psychological distance*.

**Hypothesis 5c (H5c).**  *Brand identification positively moderates the relationship between CAI and purchase intention*.

**Hypothesis 5d (H5d).**  *Brand identification positively moderates the relationship between CCI and purchase intention*.

#### 2.2.6. The Moderating Effect of Time Pressure

Existing research suggests that time pressure heightens arousal during the decision-making process, thereby decreasing the level of information retrieved in consumer memory [77]. At the same time, under time pressure, the time consumers use to gather relevant information is significantly shorter, especially information that does not carry a predisposition [48]. This may be because they need to make decisions more quickly and, therefore, become particular about selecting relevant information, focusing more on obtaining simplified information rather than taking the time to find more information [78]. Therefore, consumers pressured by time cannot comprehensively and accurately evaluate the products [79]. Consumers will now rely on heuristic rules, a quick decision-making method, to simplify the decision-making process based on experience, common sense, key product information, or other people’s opinions [80,81]. This means that when time is limited, LSE consumers will rely more on how close the products are to their construal level (experience and common sense) and whether anchors and other consumers will be active enough to provide key product information (key product information and others’ opinions). This indicates that the process of interpersonal interaction and psychological distance on purchase intention in LSE is affected by the level of time pressure. This study, thus, believes that consumers have different perceptions of time pressure and different degrees of information retrieval, and that they then pay different attention to interpersonal interaction and psychological distance. When the level of information retrieval is low (time pressure is high), the formation of consumer purchase intention is more likely to rely on direct information provided by interpersonal interaction and psychological distance. When the degree of information retrieval is high (time pressure is low), the time and level of information retrieval by consumers increases, and the formation of consumer purchase intention is more likely to rely on the information they retrieve, which reduces the influence of interpersonal interaction and psychological distance. Therefore, this study believes that time pressure positively moderates the effects of interpersonal interaction and psychological distance on consumer purchase intention. The greater the time pressure is, the stronger the influence relationship will be. The hypothesis is as follows:

**Hypothesis 6a (H6a).**  *Time pressure positively moderates the relationship between psychological distance and purchase intention*.

**Hypothesis 6b (H6b).**  *Time pressure positively moderates the relationship between CAI and purchase intention*.

**Hypothesis 6c (H6c).**  *Time pressure positively moderates the relationship between CCI and purchase intention*.

### 2.3. Research Model

**Figure 1:**
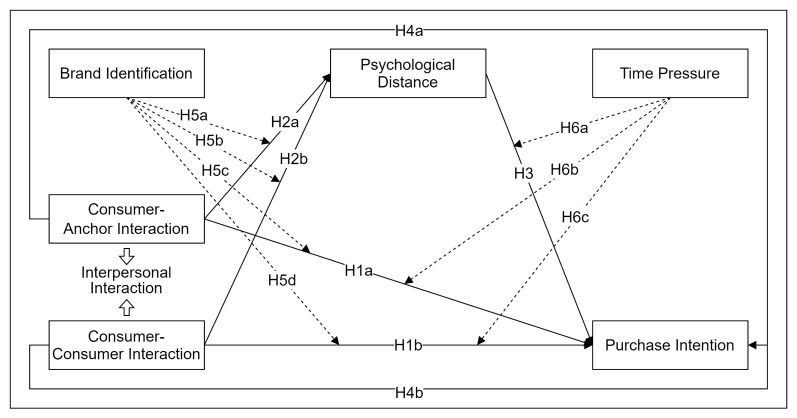
Conceptual model and assumptions.

The SOR framework assumes that the external environment can influence an individual’s emotional state and, ultimately, their behavior [82]. The framework consists of three components: stimulus (external environmental stimulus), organism (internal state), and response (final behavior). The SOR framework has been widely used in LSE-related research and effectively explains consumer behavior [16,83,84]. Existing studies have shown that the relationship between frames is also affected by LSE situational factors such as saving money and a sense of power [85,86]. In this study, interpersonal interaction is studied as stimulus “S”, psychological distance as organism “O”, and purchase intention as response “R”. Meanwhile, brand identification and time pressure are fully considered as situational factors in the context of LSE brand-exclusive and limited-time sales. Considering the above, the research model for this study was derived, as shown in Figure 1.

## 3. Methodology

### 3.1. Questionnaire

The survey employed in this research includes two primary sections: psychological perception measures and respondent characteristics. The first section involves 22 question items on consumer–anchor interaction, consumer–consumer interaction, psychological distance, brand identification, time pressure, and purchase intention. The second part contains the five commonly used question items on respondents’ characteristics. The 22 items in the questionnaire on psychological perception were based on the existing literature and were appropriately adapted to the topic of this study as shown in Table 1. Meanwhile, according to the questionnaire analysis, it was considered that the observed variables belonging to the same latent variable reflected common themes. Hence, all the variables in this study were reflective models [87,88,89]. All items in the first part of the questionnaire were measured on a 7-point Likert scale. Following a pilot survey with 20 participants, the substance of the questionnaire was adjusted and revised, using the ideas and criticisms provided by the participants to enhance the questionnaire’s comprehensibility. Pilot surveys can verify the questionnaire’s feasibility and determine whether it is clear and easily comprehensible by asking respondents to fill in questionnaires and provide feedback so that the questionnaire can be revised and adjusted as appropriate. The ultimate goal is to identify and solve problems in the pilot study so that similar problems can be avoided in the formal survey, thereby saving time and resources.

### 3.2. Data Collection

The questionnaire survey for this study was conducted from January to February 2024 in China. China has many livestreaming consumer groups and active users, thus finding respondents who met the requirements was relatively easy. The respondents of this study were consumers who had already participated in LSE shopping. To minimize sample bias to the greatest extent, we collaborated with the professional data survey platform called Wenjuanxing (www.wjx.cn, accessed on 12 February 2024), conducting a random selection of eligible respondents and offering incentives. Wenjuanxing is an experienced and authoritative data survey website that allows respondents to freely complete the questionnaire at any time and location without constraints. A specific implementation is to randomly post the questionnaire in the form of links and QR codes to anchor fan groups and live rooms and ask respondents to watch a live broadcast within the month before. To facilitate the extraction of valid questionnaires and improve the quality of data, a repetitive question was set in the questionnaire, regarding “the frequency of LSE usage each month”. Questionnaires with conflicting responses were excluded at the data collation stage. In this questionnaire study, a total of 812 questionnaires were gathered. Following the exclusion of 54 invalid questionnaires, 758 valid questionnaires were retained for analysis. Additionally, structural equation modeling was used for empirical research. This is a research method requiring that the sample size be more than 10 times the number of items measured [93], so a sample size of 758 is deemed adequate to fulfill the prerequisites of this study.

## 4. Results

This study was conducted empirically using SPSS 26 and SmartPLS 4 software. SPSS 26 was used for descriptive statistical analyses and common method bias (CMB) detection. The evaluation of measurements and structural models was conducted using SmartPLS 4 [94]. PLS-SEM is better suited for predicting linear correlations and analyzing complex models than CB-SEM. Additionally, PLS-SEM can handle a wider range of problems [95,96].

### 4.1. Demographic Profile

The respondents’ characteristics in the sample were summarized through a descriptive statistical analysis, as shown in Table 2. The results show that the percentage of females (53.4%) is higher than that of males (46.6%). This is consistent with the view that the stock of female LSE users in China surpasses that of male users [97]. At the age level, respondents in the 18–25 (39.3%) and 26–35 (36.5%) age stages accounted for the vast majority of the sample. This is not solely linked to the greater purchasing capacity of respondents within this age bracket but also aligns with the notion that individuals from the post-90s and post-00s generations have emerged as the primary user segments of LSE [98]. At the level of educational attainment, most respondents in the sample had undergraduate degrees (53.0%). At the income level, respondents with an income of CNY 3000–8000 (59.6%) occupied the highest percentage of the sample. In the survey about monthly usage, most respondents indicated that the frequency of usage was between three and six times (43.4%) in a month. The data on gender and age indicate that the demographic characteristics of this questionnaire are similar to those of Chinese LSE users, and the data distribution is reasonable and suitable for the subsequent phases of analysis.

### 4.2. Common Method Bias

In this study, a single questionnaire method was used to collect data, and CMB is possible when each variable comes from the same respondent. The presence of CMB can lead to serious bias in the results of a study [99]. Although respondents were promised anonymity and confidentiality during the questionnaire to minimize the CMB problem, before data analysis, a CMB had to be tested using Harman’s single-factor test. The analysis showed that the first factor explained only 34.631% of the variance, less than the recommended threshold of 50% [100]. The CMB was also further examined using the full-collinearity test, which showed that the VIF values for all latent variables were below the recommended threshold of 3.3 [101]. Therefore, there was no CMB in the data of this study.

### 4.3. Reliability and Validity Analysis

The results of the reliability and convergent validity analyses of the measurement models in this study are shown in Table 3. The Cronbach’s alpha coefficients ranged from 0.835 to 0.895, and the CR values ranged from 0.890 to 0.935, both greater than the recommended threshold of 0.7 [102,103]. The outer loadings (0.791–0.916) and average variance extracted values (AVE, 0.669–0.827) were greater than the recommended thresholds of 0.708 and 0.5 [95,102]. The analysis of discriminant validity was performed using two criteria. The Fornell–Larcker criterion is judged by the fact that the square root of the variable AVE is greater than the correlation coefficients of that variable with other variables. As shown in Table 4, all square roots of AVE satisfy the criterion. The criterion for heterotrait–monotrait ratio is an HTMT value below 0.85 [104]. As shown in Table 5, all the HTMT values satisfy the criterion. Therefore, the measurement model in this study has good reliability, convergent validity, and discriminant validity.

### 4.4. Collinearity Diagnostics

Before further analysis, possible multicollinearity problems in the structural model must be checked with VIF (variance inflation factor) values. The results are shown in Table 6, and all the VIF values are below the recommended threshold of 5 [95]. Therefore, there is no multicollinearity problem in this study.

### 4.5. Hypothesis Validation

**Figure 2:**
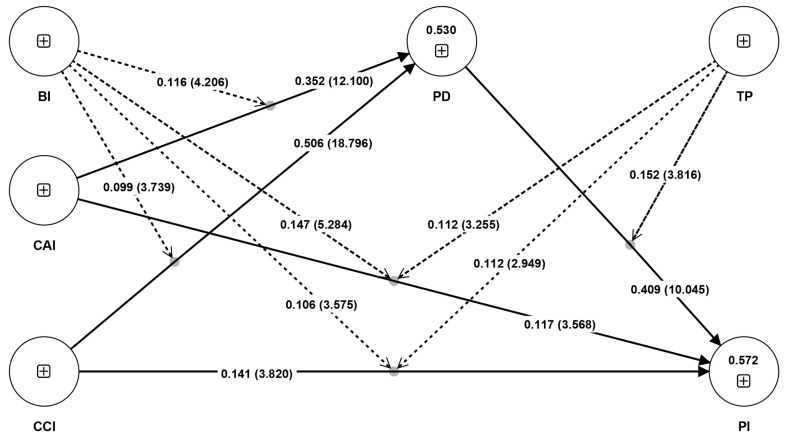
Analytical results of the model.

The path analysis was performed using bootstrapping, and the results are shown in Table 7 and Figure 2. The results of the test of direct effect showed that CAI (H1a: β = 0.117, t = 3.568, *p* < 0.001) and CCI (H1b: β = 0.141, t = 3.820, *p* < 0.001) had a significant positive effect on PI. CAI (H2a: β = 0.352, t = 12.100, *p* < 0.001) and CCI (H2b: β = 0.506, t = 18.796, *p* < 0.001) had a significant positive effect on PD. PD (H3: β = 0.409, t = 10.045, *p* < 0.001) had a significant positive effect on PI.

The results of the test for mediating effects showed that CAI (H4a: β = 0.144, t = 7.662, *p* < 0.001) and CCI (H4b: β = 0.207, t = 8.790, *p* < 0.001) had a significant mediating effect on PI through PD.

**Figure 3:**
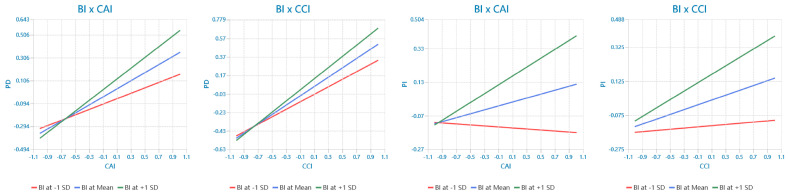
The moderating effect of brand identification.

**Figure 4:**
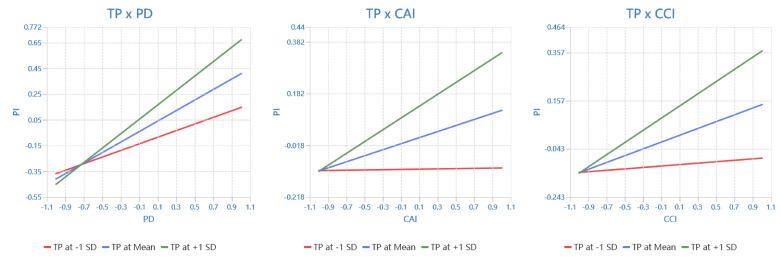
The moderating effect of time pressure.

Tests for moderating effects indicated that BI positively moderated the relationships between CAI and PD (H5a: β = 0.116, t = 4.206, *p* < 0.001) and CCI and PD (H5b: β = 0.099, t = 3.739, *p* < 0.001). Additionally, BI positively moderated the relationships between CAI and PI (H5c: β = 0.147, t = 5.284, *p* < 0.001) and between CCI and PI (H5d: β = 0.106, t = 3.575, *p* < 0.001). The interaction diagram shown in Figure 3 illustrates this point. TP positively modulated the relationship between PD and PI (H6a: β = 0.152, t = 3.816, *p* < 0.001). TP positively moderated the relationship between CAI and PI (H6b: β = 0.112, t = 3.255, *p* < 0.01) and CCI and PI (H6c: β = 0.112, t = 2.949, *p* < 0.01). The interaction diagram shown in Figure 4 illustrates this point.

### 4.6. Assessing Research Model Quality

The R^2^ and Q^2^ values were examined to assess the quality of the research model. As shown in Table 8, the R^2^ values for psychological distance and willingness to buy were 0.530 and 0.572, respectively, greater than the recommended threshold of 0.25 [105]. This indicates that the research model explained 53.0% and 57.2% of the variance in psychological distance and purchase intention, respectively. Meanwhile, the Q^2^ values exceeded the recommended threshold value of 0 [106]. Therefore, the research model has good explanatory and predictive power [107].

## 5. Discussion and Implications

### 5.1. Discussion

This study examines the impact of interpersonal interaction (CAI and CCI) on consumer purchase intention in an LSE scenario. The complex causal relationship between the variables was revealed through PLS-SEM. The statistical outcomes demonstrate that the proposed model exhibits adequate predictive capacity concerning purchase intention within the context of LSE, and all variables directly or indirectly increase consumer purchase intention. The following is a specific discussion of the findings of the study.

Interpersonal interaction (CAI and CCI) positively affects the formation of purchase intention. This suggests that the positive response of anchors to consumers and the openness of communication between consumers will positively influence consumer purchase intention for the products presented in the livestream. The range of positive responses from anchors includes addressing consumer questions, comments, and needs. Consumer-to-consumer communication then includes shopping and product experiences. This result is similar to the ones of previous studies on social commerce [108,109]. The similarity lies in the fact that the studies agree that interpersonal interaction in social commerce is a key factor influencing consumers’ purchase intentions. However, the difference lies in the fact that the division of interpersonal interaction in these studies is based on the perception of information. In contrast, the present study divides it according to different characteristics based on the differences in the main sections of the live room page. This division provides a more targeted and realistic guide to LSE and further expands the study of interpersonal interaction. The data also indicate that CCI exerts a more noticeable influence on purchase intention, implying that consumers place greater emphasis on the extent of interaction with fellow consumers. This result might be attributed to consumers perceiving information offered by non-stakeholder consumers as more objective and truthful. When they perceive sufficient communication with a trustworthy information source, it augments their purchase intention [110].

Interpersonal interaction (CAI and CCI) positively influences consumers, narrowing the psychological distance between them and the products. This implies that consumer perceptions of products become more specific during their interactions with anchors and other consumers. Their descriptions of products gradually transition from overall characteristics to localized features. This shift implies that consumers have a deeper understanding of the information about the products, which is facilitated by the high frequency and informativeness of online interactions and the positive response of anchors and other consumers. This result is similar to previous studies that have identified rich information cues and messaging as key factors in generating psychological proximity [64,111]. The difference is that these studies used pictures and AR as information sources, whereas the present study used interpersonal interaction as the starting point. The findings also demonstrate that the degree of construal level has an impact on audience judgment of psychological distance, further confirming the bidirectional causal relationship between the two [17,18].

Psychological distance significantly affects purchase intention and significantly mediates between interpersonal interaction (CAI and CCI) and purchase intention. This result indicates that interpersonal interactions can impact consumers’ psychological levels, bridging the perceived distance between products showcased in livestreaming. This creates a psychological proximity to the products, subsequently enhancing purchase intention. This is similar to some previous studies [112,113], which emphasized the mediation effect of psychological distance in influencing consumer decisions. The difference is that, while these studies used customer beliefs and information quality as the starting point of influence, this study takes interpersonal interaction as the key to trigger this series of influencing processes and divides interpersonal interaction into CAI and CCI for study. Interestingly, the data suggest that CCI is also more prominent in this influence process. This emphasizes the importance of perceived information reliability in bringing psychological distance closer and the critical role of non-stakeholders in the process.

Brand identification positively moderates the influence path of interpersonal interaction (CAI and CCI) on psychological distance and purchase intention. This outcome suggests that brand identification can enhance the influential impact of interpersonal interaction. Building a consumer sense of belonging to a brand is an effective strategy to diminish psychological distance. At the same time, if it involves a personal decision, this sense of belonging can better drive the decision. This result is similar to previous studies [114], which all emphasized the positive moderating effect of brand identification, and the reason for this result may lie in the emotional commitment and intimacy that brand identification brings to consumers. The difference is that in the study by Cachón et al., the relationship between corporate image and loyalty was used as the moderated path. In contrast, in the present study, the effect of interpersonal interaction on psychological distance and purchase intention was used as the moderated path. The study by Weitz et al. provides some mutual validation points for this study, which suggests that consumers with brand identification are more susceptible to adverse brand events and that this phenomenon arises because of the emotional commitment that accompanies brand identification [115]. Unlike several previous results, paths containing CAI were more prominent in this example. This may occur because the employment relationship between the anchor and the brand manufacturer in LSE makes the anchor represent the brand to a certain extent, which makes consumers with brand identification pay more attention to the interaction with the anchor. This discovery expands previous studies on the moderating influence of brand identification and provides a strong reference for understanding consumer psychology in the context of brand-exclusive LSE.

Time pressure positively moderates the path of influence of psychological distance and interpersonal interaction (CAI and CCI) on purchase intention. This finding suggests that time pressure can augment the influential impact of both psychological distance and interpersonal interaction. It also implies that time pressure can better drive individual decision-making under certain conditions. This finding aligns with several prior studies suggesting that time pressure prompts consumers to bear opportunity costs [116,117], which increases purchase intention. However, it also differs from the results of some previous studies, and these research perspectives suggest that negative emotions such as anxiety caused by time pressure can affect the consumer’s shopping experience [22,118], thus reducing purchase intention. By combing through the existing literature, this study concludes that there are two main reasons for the variety in results. First, the literature holding an opposing viewpoint suggests that furnishing consumers with product information and enhancing interactive experiences effectively mitigate the adverse impacts of time pressure [22]. This highlights the disparity in the outcomes of the current study, which specifically investigates the role of time pressure in interactions. The present study is, therefore, a validation of the improvement strategies proposed in previous research, demonstrating the different moderating effects of time pressure in specific conditions. Furthermore, the current literature indicates that experience plays a crucial role in the varied effects of time pressure, as experienced consumers tend to elevate their purchase intention under time constraints, whereas less experienced consumers exhibit the opposite trend [119]. This experience is related to the format of the livestreaming and the measurement criteria of the relevant variables. In CAI, anchors tend to communicate with consumers with hands-on usage experience as the basis of the live content. In CCI, consumers use experiences such as shopping experiences and product experiences as the basis for communication [13,31]. In psychological distance, experiences such as the degree of truthfulness and specificity are used as measures [69]. Hence, heightened levels of interpersonal interaction and psychological distance intensify consumer experiential perception of products showcased in livestreaming sessions, even when such experiences come from information provided by others. This perception of experience partially counteracts the adverse effects of time pressure. This finding expands upon prior research regarding the moderating impact of time pressure and offers a robust reference for understanding consumer psychology in the context of time-limited LSE.

### 5.2. Implications

#### 5.2.1. Theoretical Implications

This study provides some theoretical contributions to the field of LSE marketing. First, this study provides new insights into how interpersonal interaction expressions are delineated in the context of LSE. Most existing studies on LSE interactions have been conducted using a single dimension [9,14,15,16]. This single-dimensional division does not consider the actual events in the live room. It ignores the interaction between people with different identities brought about by the live display and consumer message areas. This makes the existing research pay insufficient attention to the differences between interactions. Interpersonal interaction with this kind of dimension division according to different identities is very rare. This provides researchers with new perspectives and practitioners with more focused recommendations.

Second, the results of this study also provide more relevant insights into understanding consumer purchase intention in LSE. Most existing studies on LSE have focused on the general sales model [56,84,120], ignoring the importance of brand-exclusive and limited-time sales as the main forms of LSE. This makes the results of many studies not entirely applicable to real-world situations. Brand identification is a core element of brand relationships [38], and time pressure is an important situational variable in time-limited sales [51]. Therefore, this study can fully consider the actual situation and incorporate brand identification and time pressure into the research model, which provides a new perspective for understanding consumers’ behavioral patterns and decision-making processes in the LSE environment.

Finally, this study confirms the suggestions made by Peng et al. in a study related to time pressure. Peng et al., concluded that time pressure reduces the shopping experience and, ultimately, the purchase intention of consumers and suggested that increased interaction regarding consumer counseling might be a way to reduce the negative effects of time pressure [22]. This study confirms empirically that in an LSE environment, interaction not only helps to alleviate the negative impact of time pressure on consumer behavior but also makes consumers more willing to participate in purchase activities. This study fills the gap in previous research on how time pressure affects purchase intention in an interactive environment and is important for expanding consumer behavior models.

#### 5.2.2. Practical Implications

This study has some practical implications for managers and practitioners of LSE platforms. The basis for the practical implications is the recognition of the important contribution of communication about products and emotions, as enabled by CAI and CCI, to consumers’ purchase intentions in LSE frameworks. Both emphasize the critical role of efficient collaboration and engagement enabled by the characteristics of livestreaming in reducing the psychological distance between consumers and products. The important implications of CAI and CCI provide actionable guidance for LSE platforms.

First, relevant practitioners should uncover the key role of CAI in livestreaming shopping for consumers. In addition to proper training for the anchors to improve their interactive performance to ensure maximum utilization of CAI, technical metrics can be used to extract and summarize keywords of the live chat in the live room. It is convenient for the anchor to understand what consumers most urgently want to know the first time, making the interaction more targeted. Simultaneously, considering the bidirectional causal relationship between psychological distance and construal level, the anchor’s description of the products in the livestreaming should be carried out for the surface and local features. This expression can make the products more concretized in the consumer’s mind and bring closer the psychological distance between the two.

Second, managers of LSE platforms should realize the important role of CCI and improve the efficiency and effectiveness of interaction between consumers through innovative interaction. For example, a prominent logo can be added after the IDs of consumers who have previously purchased the product. Such a logo can attract other consumers to pay more attention to their reviews, comments, or suggestions and promote more active communication and information sharing among consumers.

Third, given the significant impact of brand identification on consumer purchasing decisions, relevant practitioners should carry out the necessary publicity and promotional activities of the relevant brand before the official livestreaming. Such promotional activities should make full use of the corresponding technical metrics to accurately push to the consumer group that meets the user profile of the brand to attract consumers who already have brand identification or potentially have a sense of identification. They should also utilize the facilitative impact of brand identification on interpersonal interaction to influence consumer psychology and behavior, thereby enhancing the effectiveness and conversion rate of marketing campaigns.

Fourth, this study’s findings indicate that a moderate level of time pressure can favorably impact customers’ buying choices, in certain circumstances. However, relevant practitioners should understand that sufficient interaction in livestreaming is crucial for the time pressure effect to be positive and a lack of sufficient interaction may reduce the promotional effect of time pressure. Therefore, when implementing a time-limited sales strategy, paying attention to the interaction during the livestreaming is particularly important. If the interaction activity is found to be low, relaxing the time limit may be more in line with the needs of consumer decision-making to avoid time pressure adversely affecting the consumer experience.

Furthermore, considering the formulation of the interpersonal interaction–psychological distance–purchase intention framework, this study emphasizes the importance of the government and relevant regulatory agencies to enhance the oversight of LSE platforms. These regulatory measures should require platforms to provide authentic and reliable interpersonal interaction environments to prevent unscrupulous merchants from utilizing falsely advertised interpersonal interaction methods that reduce the psychological distance between the consumer and the products, thereby triggering fraudulent behaviors.

## 6. Conclusions, Limitations, and Future Research

This study aims to explore the mechanism of interpersonal interaction (CAI and CCI) on consumer purchase intention in LSE scenarios and establishes a theoretical model of interpersonal interaction–brand identification–psychological distance–time pressure–purchase intention. The findings indicate that interpersonal interaction plays a crucial role in shaping consumer psychological distance and purchase intention toward livestreaming products. On the other hand, psychological distance has a mediating role between interpersonal interaction and purchase intention. The findings indicate that interactions among individuals with different populations within LSE favorably influence consumer decision-making. A pivotal element in this process is the capacity of interaction to diminish psychological distance. Furthermore, the study uncovers the affirmative moderating impact of brand identification and time pressure within this sequence of effects. This study offers novel insights and recommendations from a multi-theoretical standpoint within the framework of LSE.

This study has some limitations that need to be addressed in future research. First, purchase intention may not comprehensively mirror real consumer purchasing actions. Therefore, future research will consider longitudinal case studies to understand how purchase intentions and consumer behavior change over time. An in-depth qualitative study will also be conducted through interviews to gain a more comprehensive understanding of the experience of consumers using LSE platforms. Second, the model verified the direct, mediating, and moderating effects between the variables but ignored the moderating effects of brand identification and time pressure on the mediating relationships. Therefore, future research will validate these paths to better understand the mechanisms that influence consumer purchase intention. Third, since most respondents were from China, the model’s applicability may be limited by LSE development and cultural diversity variations. Future research will collect questionnaires in regions with varying degrees of LSE development and consumer culture to further validate the model’s validity and establish cross-cultural comparisons. Finally, this study only considered the factor of interpersonal interaction in LSE and ignored the possible interactivity of the software interface. Therefore, relevant variables will be added in future studies to provide a more comprehensive understanding of the impact of livestreaming interactions on consumer decision-making behaviors.

## Figures and Tables

**Table 1:** Measurement.

Constructs	Items	Sources
CAI	The anchor can answer my specific questions clearly and quickly.	Ma et al. Ma et al. [13,31]
	The anchor can interact with me on product-related information.	
	The anchor’s responses are closely related to my comments.	
	The anchor’s response can fulfill my needs.	
CCI	I can exchange shopping experiences with other consumers.	
	I can exchange product experiences with other consumers.	
	I can fully communicate with other consumers.	
	I can get a lot of product-related information from other consumer comments.	
Psychological Distance (PD)	This product is very concrete in my mind.	Sun et al. [69]
	This product is very real in my mind.	
	This product is very close to me in my mind.	
Brand Identification (BI)	This brand is like a part of me.	Yeh et al. Kumar et al. [71,90]
	The brand has a lot of personal meaning for me.	
	I have a strong sense of belonging to the brand.	
	When someone compliments the brand, it feels like a compliment to me.	
Time Pressure (TP)	No time pressure/Too much time pressure.	Suri et al. [91]
	More than adequate time available/Not adequate time available.	
	Not in need of more time to consider this purchase decision/In need of more time to consider this purchase decision.	
Purchase Intention (PI)	I would consider purchasing these products.	
	There is a high probability that I will purchase the products.	Peng et al. [22]
	I will purchase these products soon.	Wang [92]
	I would like to purchase the products if I have enough time, energy, and money.	

**Table 2:** Descriptive Statistics (N = 758).

	Items	Frequency	Proportion
Gender	Male	353	46.6%
	Female	405	53.4%
Age (in years)	18–25	298	39.3%
	26–35	277	36.5%
	36–45	122	16.1%
	>45	61	8.0%
Education	High school or below	122	16.1%
	Three-year college	183	24.1%
	Undergraduate	402	53.0%
	Postgraduate or above	51	6.7%
Monthly income (CNY/Yuan)	<3000	97	12.8%
	3000–8000	452	59.6%
	8000–13,000	163	21.5%
	>13,000	46	6.1%
Number of monthly uses	<3	162	21.4%
	3–6	329	43.4%
	7–10	177	23.4%
	>10	90	11.9%

**Table 3:** Reliability and validity analysis.

Constructs	Item	Factor Loadings	Cronbach’s Alpha	CR	AVE
CAI	CAI1	0.868	0.858	0.904	0.702
	CAI2	0.835			
	CAI3	0.830			
	CAI4	0.817			
CCI	CCI1	0.871	0.835	0.890	0.669
	CCI2	0.799			
	CCI3	0.791			
	CCI4	0.808			
Psychological Distance (PD)	PD1	0.899	0.895	0.935	0.827
	PD2	0.916			
	PD3	0.913			
Brand Identification (BI)	BI1	0.846	0.840	0.892	0.675
	BI2	0.822			
	BI3	0.819			
	BI4	0.798			
Time Pressure (TP)	TP1	0.896	0.869	0.919	0.792
	TP2	0.898			
	TP3	0.875			
Purchase Intention (PI)	PI1	0.896	0.891	0.925	0.755
	PI2	0.844			
	PI3	0.890			
	PI4	0.843			

**Table 4:** Discriminant Validity (FORNELL).

	CAI	CCI	PD	BI	TP	PI
CAI	0.838					
CCI	0.348	0.818				
PD	0.526	0.622	0.909			
BI	0.191	0.332	0.280	0.821		
TP	0.201	0.251	0.247	0.215	0.890	
PI	0.395	0.454	0.625	0.292	0.241	0.869

**Table 5:** Discriminant Validity (HTMT).

	CAI	CCI	PD	BI	TP	PI
CAI						
CCI	0.409					
PD	0.599	0.717				
BI	0.225	0.389	0.316			
TP	0.234	0.292	0.280	0.251		
PI	0.452	0.523	0.699	0.333	0.273	

**Table 6:** VIF Value of the Inner Model Matrix.

	CAI	CCI	PD	BI	TP	PI
CAI			1.150			1.420
CCI			1.273			1.881
PD						2.196
BI			1.151			1.196
TP						1.140
PI						
BI × CAI			1.099			1.195
BI × CCI			1.153			1.229
TP × PD						1.991
TP × CAI						1.395
TP × CCI						1.853

**Table 7:** Hypothesis testing.

Paths	Hypotheses	Path Coefficients β-Values	*t*-Values	*p*-Values	Confidence Interval		Decision
					2.5%	97.5%	
Direct effects							
CAI → PI	H1a	0.117	3.568	0.000	0.052	0.182	Supported
CCI → PI	H1b	0.141	3.820	0.000	0.069	0.215	Supported
CAI → PD	H2a	0.352	12.100	0.000	0.297	0.410	Supported
CCI → PD	H2b	0.506	18.796	0.000	0.451	0.557	Supported
PD → PI	H3	0.409	10.045	0.000	0.332	0.487	Supported
Mediation effects							
CAI → PD → PI	H4a	0.144	7.662	0.000	0.109	0.184	Supported
CCI → PD → PI	H4b	0.207	8.790	0.000	0.162	0.254	Supported
Moderating effects							
BI × CAI → PD	H5a	0.116	4.206	0.000	0.062	0.169	Supported
BI × CCI → PD	H5b	0.099	3.739	0.000	0.044	0.150	Supported
BI × CAI → PI	H5c	0.147	5.284	0.000	0.092	0.201	Supported
BI × CCI → PI	H5d	0.106	3.575	0.000	0.045	0.163	Supported
TP × PD → PI	H6a	0.152	3.816	0.000	0.072	0.229	Supported
TP × CAI → PI	H6b	0.112	3.255	0.001	0.048	0.182	Supported
TP × CCI → PI	H6c	0.112	2.949	0.003	0.037	0.186	Supported

**Table 8:** R^2^ value and Q^2^ value.

	R^2^	Q^2^ Predict
Psychological Distance (PD)	0.530	0.518
Purchase Intention (PI)	0.572	0.444

## Data Availability

Data available upon request.
